# Automatic Meningioma Segmentation and Grading Prediction: A Hybrid Deep-Learning Method

**DOI:** 10.3390/jpm11080786

**Published:** 2021-08-12

**Authors:** Chaoyue Chen, Yisong Cheng, Jianfeng Xu, Ting Zhang, Xin Shu, Wei Huang, Yu Hua, Yang Zhang, Yuen Teng, Lei Zhang, Jianguo Xu

**Affiliations:** 1 Department of Neurosurgery, West China Hospital, Sichuan University, No. 37 GuoXue Alley, Chengdu 610041, China; chaoyuechen01@gmail.com (C.C.); chengyisong2020@wchscu.cn (Y.C.); DrZhangTing0604@163.com (T.Z.); dryangzhang66@gmail.com (Y.Z.); yuenteng01@gmail.com (Y.T.); 2 Department of Critical Care Medicine, West China Hospital, Sichuan University, No. 37 GuoXue Alley, Chengdu 610041, China; 3 Department of Neurosurgery, Third People’s Hospital of Mianyang/Sichuan Mental Health Center, No. 109 Jianan Road, Mianyang 621000, China; xujianfeng3@sohu.com; 4 College of Computer Science, Sichuan University, Chengdu 610065, China; shuxin@stu.scu.edu.cn (X.S.); weihuang@stu.edu.scu.cn (W.H.); 2020223040084@stu.scu.edu.cn (Y.H.)

**Keywords:** meningiomas, deep-learning technology, magnetic resonance imaging, segmentation, grading prediction

## Abstract

The purpose of this study was to determine whether a deep-learning-based assessment system could facilitate preoperative grading of meningioma. This was a retrospective study conducted at two institutions covering 643 patients. The system, designed with a cascade network structure, was developed using deep-learning technology for automatic tumor detection, visual assessment, and grading prediction. Specifically, a modified U-Net convolutional neural network was first established to segment tumor images. Subsequently, the segmentations were introduced into rendering algorithms for spatial reconstruction and another DenseNet convolutional neural network for grading prediction. The trained models were integrated as a system, and the robustness was tested based on its performance on an external dataset from the second institution involving different magnetic resonance imaging platforms. The results showed that the segment model represented a noteworthy performance with dice coefficients of 0.920 ± 0.009 in the validation group. With accurate segmented tumor images, the rendering model delicately reconstructed the tumor body and clearly displayed the important intracranial vessels. The DenseNet model also achieved high accuracy with an area under the curve of 0.918 ± 0.006 and accuracy of 0.901 ± 0.039 when classifying tumors into low-grade and high-grade meningiomas. Moreover, the system exhibited good performance on the external validation dataset.

## 1. Introduction

Meningioma is recognized as one of the most common intracranial neoplasms, accounting for 30% of primary intracranial lesions, with an annual incidence of 5/100,000 [1,2]. Based on their biological behaviors, the lesions can be categorized as either low-grade lesions (grade I meningioma), which are correlated with better survival prognosis, or high-grade lesions (grade II and grade III meningiomas), which are correlated with aggressive behaviors along with increased recurrence risk following treatment [2,3,4]. When intervention is required, surgery is usually the optimal choice, considering that the majority of patients can be cured with surgery alone, especially for grade I tumor patients with favorable locations. Radiotherapy approaches are also recommended, especially for high-grade meningiomas, to increase local control when surgery alone seems insufficient [5,6]. The high spatial resolution and excellent soft-tissue resolution of MRIs provide the necessary information for clinicians to facilitate treatment decisions [5]. The image patterns of tumors on MRIs are usually described as solitary round tissues that are in close contact with the dura mater, with apparent/homogeneous enhancement after gadolinium injection. The thickening of the dura mater around the tumor, known as the dural tail, is also present in contrast-enhanced T1 images with contrast injection [7]. Empirically, aggressive meningiomas usually present with heterogeneous patterns accompanied by irregular shapes and larger volumes, when compared with benign meningiomas [8]. However, the consensual radiological criteria that can be applied to distinguish meningiomas of different grades accurately are not yet clear [5].

The patient diagnostic assessment usually focuses on two points: tumor progression, which indicates that an automated evaluation of tumors should improve image reading, and grading prediction, which is significantly relevant to treatment strategy and patient prognosis. In the current study, we established an operable MRI-based system for meningioma assessment using deep-learning technology. The system comprises an automatic detection module, a 3D-rendering module, and an automatic grading prediction module. We hope that it can help clinicians make individual surgery plans, evaluate the risk of surgery, and predict patient outcomes.

## 2. Materials and Methods

### 2.1. Dataset

Two datasets from two institutions were used in this study. We chose contrast-enhanced images of the three-dimensional magnetization-prepared rapid gradient-echo (MPRAGE) sequence in the following research, given that the segmentation required clear presentation on the tumor boundary and location. Moreover, a slice thickness of 1 mm for this sequence was sufficiently thin to meet the requirements for 3D spatial reconstruction.

In the first dataset, the researchers retrospectively collected preoperative MRI scans of 625 patients in the neurosurgery department between 2008 and 2018. All patients underwent surgery and were pathologically diagnosed with meningioma. After an initial evaluation, some patients were excluded from the study for the following reasons: (1) images with noticeable motion artifacts; (2) untraceable treatment history in other hospitals (such as radiotherapy or surgery); and (3) recorded intracranial disease history (such as subarachnoid hemorrhage, cerebral infarction, etc.). Two scanners performed standard MR scans in the radiology department, and the detailed protocols are summarized as follows:

3.0T Siemens Trio Scanner, MPRAGE sequence, parameters as: TR/TE/TI = 1900/2.26/900 ms, flip angle = 9°, 176 axial slices with thickness = 1 mm, axial FOV = 25.6 × 25.6 cm^2^ and data matrix = 256 × 256.

3.0T GE SIGNA MRI scanner, MPRAGE sequence, parameters as: TR/TE = 8.5/3.4 ms, flip angle = 12°, 156 axial slices with thickness = 1 mm, axial FoV = 24 × 24 cm^2^, and data matrix = 512 × 512.

Contrast-enhanced images were acquired following the injection of gadopentetate dimeglumine (dose: 0.1 mmol/kg). Based on these principles, 97,500 images of 625 patients were collected as the image dataset. In addition, the clinical characteristics of the patients were collected, as listed in Table 1.

The second dataset included 2808 images of 18 low-grade meningioma patients from another institution, whose MRI scans were obtained with a Siemens MAGNETOM Symphony 1.5T MRI System (7 patients) or Siemens MAGNETOM Skyra 3T MRI System (11 patients). Unfortunately, because of institutional data protection laws, the data had been desensitized, and all information related to patient privacy or MR protocols were removed. Therefore, we were unable to share more detailed MR sequence parameters or patient characteristics. Considering the small cohort of this dataset, it served as the external validation group for this research.

This study was approved by the institutional review boards of West China Hospital, Sichuan University, who granted a waiver of informed consent (2021-S-851).

### 2.2. Human Segmentation

The tumor images were manually segmented by two experienced neurosurgeons in a consensus reading using the LIFEx software [9]. Following the software instructions, the surgeons were asked to accurately delineate the region of interest (ROI) along the tumor boundary slice by slice on axial view images. The adjacent structure invasion and peritumoral edema band were separated from the ROI by the different enhanced patterns in contrast enhancement, whereas vessels and necrosis inside the tumors were included in the ROI. When the authors were in disagreement with each other, they recorded it and consulted senior neurosurgeons and senior radiologists. Finally, the labels were checked by the corresponding authors to make final decisions.

### 2.3. Establishment of the Deep-Learning System

**Figure 1:**
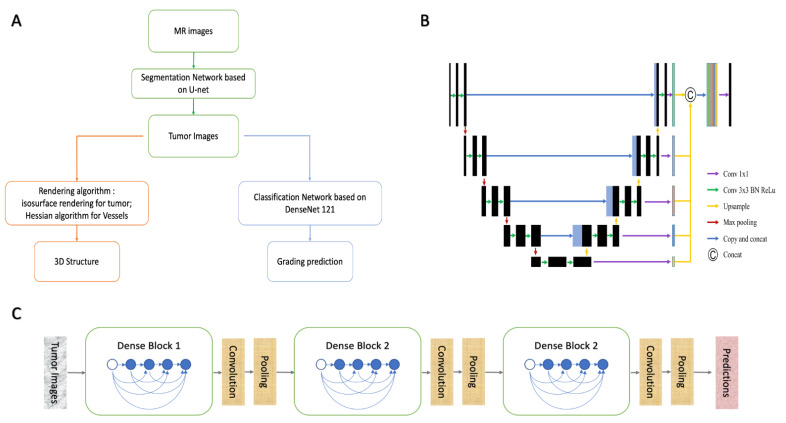
System structure designed in this research. (**A**). The cascade structure of system; (**B**). Network architecture of U-Net; (**C**). Network architecture of Dense-Net.

As mentioned above, the system was established with a cascade structure consisting of three modules (Figure 1A). The parameters of the models or algorithms were set as follows.

#### 2.3.1. Automatic Detection and Segment Model

The segment model was designed with a modified U-Net, a convolutional deep neural network (CNN), showing promising ability in segmentation tasks of medical images (Figure 1B) [10]. The inputs could be selected as a 3D stack or a single 2D image depending on the task needs. To compare their performances, we established two models with both 2D-U-net and 3D-U-net, and the parameters were set as follows:

The basic structures of the 2D-U-Net architecture consist of cascading layers of learnable convolutional filters. The configuration included five down-sampling steps, which reduced the 512 × 512 input images into 16 × 16 × 196 representations, and five up-sampling steps, which up-sampled the representations into 512 × 512 × 6 outputs. As in down-sampling steps, each layer consisted of two consecutive 3 × 3 convolutions, following a normalization of values to the range of −1 to +1 with a hyperbolic tangent (tanh) activation function. The features of down-sampling were max-pooled using a 2 × 2-pixel kernel. As in up-sampling steps, operations were performed via 2 × 2 nearest-neighbor interpolation and two tanh-activated convolutional layers. The final layer consisted of a convolution with a 1 × 1 kernel and a sigmoid function to output the score for the tissue classes. The segmentation of tumor tissue was achieved by selecting the class with the highest score. We experimentally determined the optimal hyperparameters and randomly initialized the network parameters [11]. The batch size of the sections was 48, and the learning rate was constant at 0.005 [12]. Segment performances of the training and validation sets were evaluated using categorical cross-entropy. The model was trained using a constant cycle of 80.

The structure of 3D-U-Net was similar to that of 2D-U-Net, the difference being the involvement of spatial information in the input images. Specifically, down-sampling reduced the 512 × 512 × 10 input images into 16 × 16 × 196 × 8 representations, with two consecutive 3 × 3 × 3 convolutions and max-pooling by a 2 × 2 × 2-pixel kernel. Up-sampling recovered the representations into 512 × 512 × 6 × 8 outputs, with 2 × 2 × 2 nearest-neighbor interpolation and two tanh-activated convolutional layers. The batch size of the sections was four, and the learning rate was 0.005 at decay every five rounds. The model of 3D-U-Net was trained at a constant cycle of 40.

The 5-fold cross validation was adapted to verify the performances of the models. Manual segment labels were used as the ground truth. The performances of the two models were analyzed using the following parameters: dice score, Jaccard score, true-positive fraction (TPF), false-positive fraction (FPF), and calculation time. These models were programmed using the Python programming language, and the hardware platform was an NVIDIA Tesla P100 data center accelerator.

#### 2.3.2. Automatic 3D-Rendering Module

The reconstruction module consisted of two parts.

The first part focused on the demonstration of the tumor, for which we proposed an automatic rendering system focusing on visualizing brain tissue and tumor body spatially. Tumor image labels were produced by the segmentation model, and brain image labels were extracted using a CNN model reported previously [13]. Then, the segmentations and image volumes were introduced into a 3D Slicer for construction using a composite with shading technology [14].

The second part focused on the relationship between tumors and intracranial vessels, considering that the principle of surgical intervention for meningioma was safe maximal tumor removal. An insight toolkit (Itk) reader was introduced, followed by importing into a visualization toolkit (vtk) converter. We employed the isosurface-rendering algorithm to reconstruct the tumor spatially and the maximal intensity projection (MIP) algorithm to reconstruct the intracranial vessels. This part was written in the C++ programming language.

#### 2.3.3. Grading Prediction Model

The grading model was constructed using classic DenseNet 121, a network with promising performance in classification tasks [15]. In contrast to previous CNN networks, DenseNet introduced direct connections from any layer to all subsequent layers. The basic structures of this network are dense blocks and transition layers (Figure 1C). Each dense block contained batch normalization (BN), rectified linear unit (ReLU), and convolution layers (convolution kernel: 3 × 3), while the selected features were transferred into transition layers to achieve a pooling operation. In the last layer, the tumor grading classification was determined using a neuron designed with a sigmoid activation function. The optimal hyperparameters were determined experimentally, and the parameters of the network were initialized by pre-training in ImageNet. The batch size was set to 64, and the learning rate was 0.0005 at decay every five rounds. The classification model was trained using a constant cycle of 200.

DenseNet inputs could be either completed images (Plan A) or segmented images (Plan B). In the current study, we evaluated both plans to select the optimal one. The area under the curve (AUC), accuracy, FPR, TPR, and calculation time were adapted to evaluate the classification performance. Grading models were written in the Python programming language, operated in the NVIDIA Tesla P100 data center accelerator.

### 2.4. System Test on the External Dataset

To verify the robustness of previous research, we tested the system on the second dataset from another institution to explore the performance of the modules. As stated, the MR images were acquired from two platforms with different protocols in the second institution. One of the platforms was a 3.0T MRI scanner, while the other was a 1.5T MRI scanner with which the images were of lower quality. Because all patients in the second dataset were diagnosed with low-grade meningioma, the test results of the grading prediction modules seemed unreliable. Therefore, the test focused on the segmentation modules and 3D rendering modules.

## 3. Results

### 3.1. Tumor Segmentation and Reconstruction

**Figure 2:**
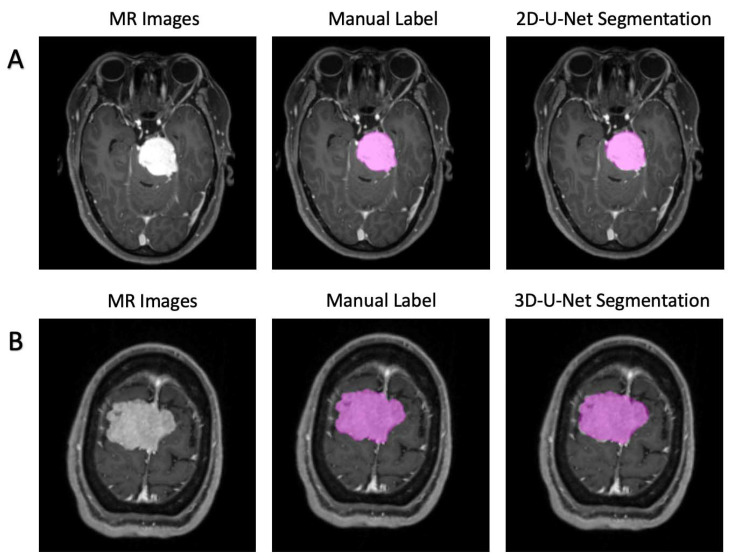
Two examples of the segmentation predicted by U-Net. (**A**). 2D-U-Net architecture; (**B**). 3D-U-Net architecture.

**Figure 3:**
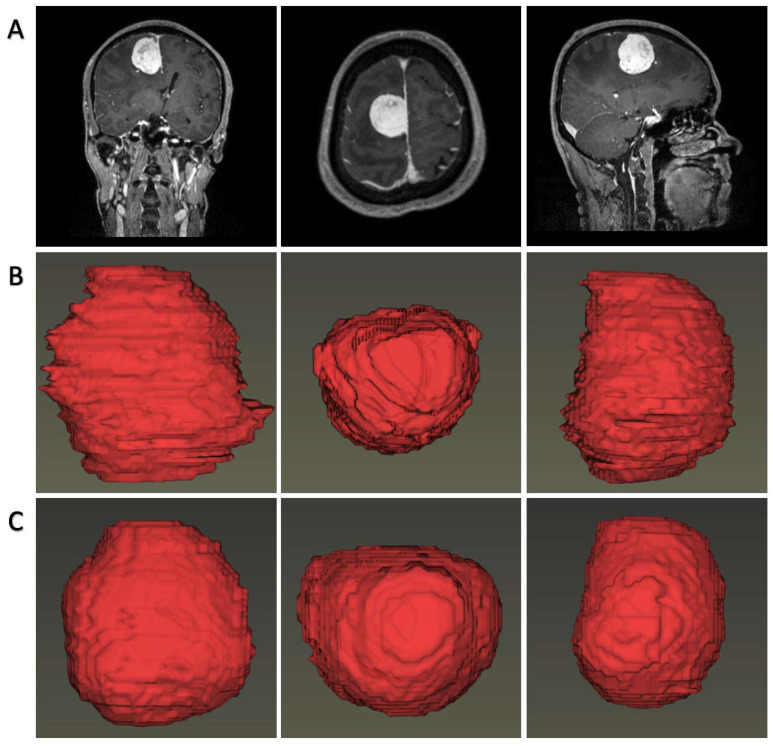
Visualized tumor reconstructions by automatic predicted labels and human-noted labels. (**A**). original MR images; (**B**). tumor rendering with human-noted labels; (**C**). tumors rendering with automatic predicted labels.

In most cases, both 2D-U-Net and 3D-U-Net can accurately segment tumors on axial images. Figure 2 represents the predicted section examples from the models’ tumor predictions in comparison with the manual segmentations. Specifically, the performances for the 2D-U-net network in the validation groups were: dice = 0.920 ± 0.009, Jaccard = 0.851 ± 0.016, TPR = 0.999 ± 000, FPR = 0.000 ± 0.000, and calculation time = 70 ms/image. Additionally, for 3D-U-net, the results were: dice = 0.873 ± 0.020, Jaccard = 0.774 ± 0.015, TPR = 0.999 ± 0.002, FPR = 0.000 ± 0.000, and calculation time = 390 ms/image. The 3D visualization of our detections and ground-truth segmentations are shown in Figure 3.

However, the models were unable to feasibly segment the tumor section regardless of the architecture of 2D-U-net or 3D-U-net under the following circumstances: A. in images where the tumor was adjacent to thickening of the dura mater; B. meningiomas that were located in the anterior skull base; C. form-complicated tumors, such as high-grade meningiomas with apparent necrosis inside the lesions (Supplement Figure S1).

### 3.2. Automatic 3D-Reconstruction of Tumors and Vessels

**Figure 4:**
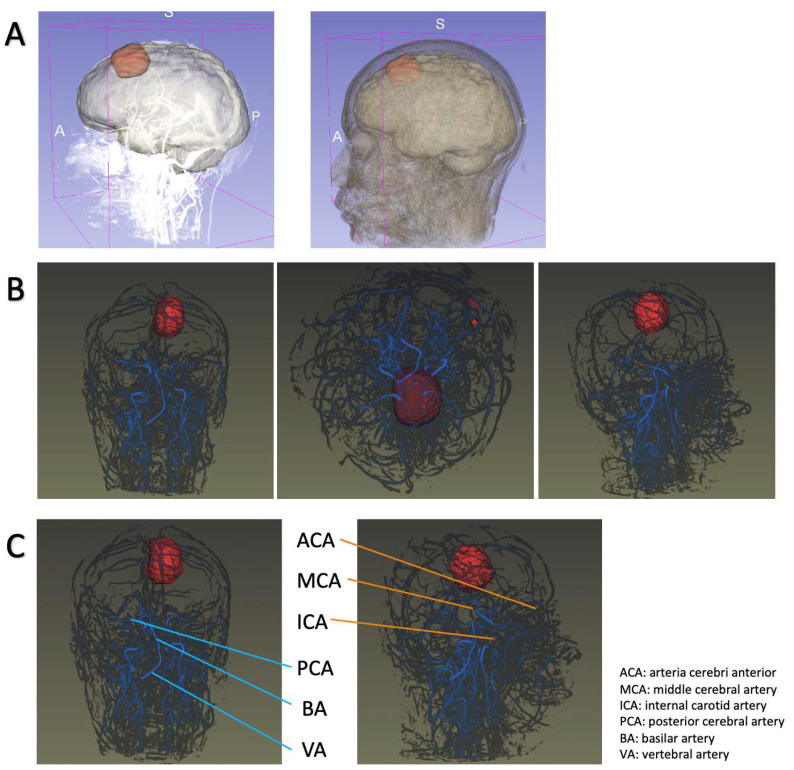
Intellectual system design. (**A**). meningioma demonstration system; (**B**). automatic renderings of tumors and intracranial vessels; (**C**). recognition of intracranial vessels.

In contrast to previous research, we presented our results as a system for demonstration and evaluation rather than a single task. We used the 2D-U-net model for segmentation, not only because of its high dice coefficient but also because of the non-existent bias suggested by the Bland–Altman plots (Supplementary Figure S2). With accurate segmented images, the isosurface-rendering algorithm delicately reconstructed tumor bodies with sophisticated surfaces and clearly defined structures. In addition, the vessels were also spatially represented, and the major arteries (such as the middle cerebral artery and basilar artery) were recognizably constructed (Figure 4). We believe that this module could potentially be severed in the pre-operation demonstration in the future.

### 3.3. Performances of Grading Models

Generally, grading models have a promising ability to classify low-grade meningiomas from high-grade meningiomas. The high performance of the segmentation network laid a brilliant foundation for the classification network, which proved that Plan B represented significantly improved classification performance compared with Plan A. For Plan A, AUC, accuracy, FPR, TPR, and calculation time for the validation group were 0.837 ± 0.034, 0.813 ± 0.086, 0.833 ± 0.052, 0.800 ± 0.033, and 3.08 ms/image, respectively. For models trained with Plan B, the AUC, accuracy, FPR, and TPR were 0.918 ± 0.006, 0.901 ± 0.039, 0.925 ± 0.011, 0.925 ± 0.011, and 3.01 ms/image, respectively.

### 3.4. Robustness of the System on Different Datasets

**Figure 5:**
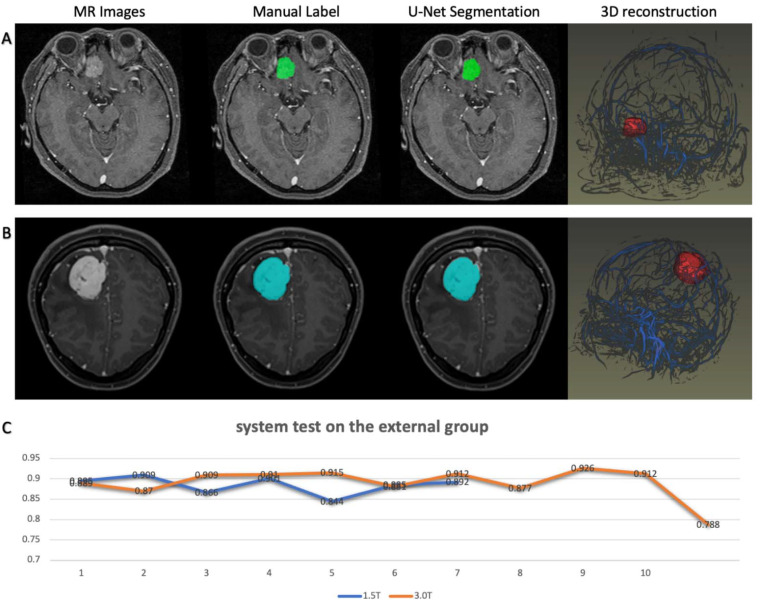
System performances on the external validation group. (**A**) 1.5T MR scanner platform; (**B**) 3.0T MR scanner platform; (**C**) Dice score of each patient in Table 1: Characteristics of patients involved in the experimental dataset.

The robustness of the current research was tested on the second dataset. The models showed better compatibility with the 3.0T MR scanner than with the 1.5T MR scanner. As shown in Figure 5, the segmentations generated by the system were highly consistent with the ground-truth labels for the second dataset, indicating potential feasibility in clinical applications.

## 4. Discussion

In this study, we proposed a meningioma assessment system based on routine MR scan images combined with deep-learning technology. The system was fully automated and capable of detecting tumors on MRIs, displaying tumor structure/intracranial blood vessels, and predicting tumor grade. It is reasonable to consider that the system was mature and stable, as the models were trained with the largest sample size so far and the system was tested on an external dataset. Given that MRI is the necessary radiological examination for intracranial tumors, our system could be potentially utilized in clinical work to facilitate treatment decisions without financially burdening patients.

Our research showed that deep learning is a feasible technology for meningioma assessment by neurosurgeons and neuro-oncologists. Tumor rendering can improve clinical assessment in routine imaging approaches, as it might allow for enhanced detection, improved monitoring, and precise surgery planning [16]. In addition, automated 3D rendering allows for standardized tumor volume definition as it avoids inter- and intra-rater subjective assessment, which has been demonstrated as high [17]. Combined with the grading prediction module, the system can be used for tumor progress monitoring and assessment. For neurosurgeons, a system with vessel rendering could also be potentially applied for operation planning [5]. However, the rendering module still requires modifications at two points. First, manual corrections of predicted labels were still needed to make a more precise delineation where the segmentation module did not work well. The other was that vessel rendering still needs to be improved to automatically recognize the specific arteries and show/hide a particular artery when the clinician needs. We aim to solve these problems in future studies.

The system could also be used to enhance the pre-surgery communication between neurosurgeons and patients, which has been reported to be clinically significant but difficult. Indirect visualization of intracranial tumors using MR images is difficult for patients, as it requires professional knowledge or skill obtained from years of practice. Ineffective communication can lead to patients failing to truly understand their condition, followed by arguments, followed by complaints. Previous research reported that neurosurgeons were much more likely to generate unsolicited patient complaints than other surgical specialties, and 71.6% of neurosurgeons had at least one complaint during their study [18]. Rendering structures could provide better, clearer demonstrations for patients by providing basic information about the tumor, including size, location, shape, and grading prediction. It is reasonable to consider that this technology has great potential in clinical practice.

The results indicated that the high performance of this system, regardless of the automatic reconstruction modules or grading prediction modules, primarily relied on accurate segmentation by the U-net model. Automatic detection and segmentation of meningiomas were performed in a previous study, and with good performances [19]. There were two significant improvements between the previous model and our segmentation models [19]. First, our models were entirely trained on the large sample meningioma dataset, while the previous work employed pre-training, an implemented option that may alleviate the limitation of small datasets on the glioma dataset [20,21]. Second, our models were “end-to-end” methods without requiring extra pipelines for extensive image post-processing and registration. These points improved the segment performance and reduced the calculation time. Above all, it is reasonable to consider that our models outperformed previous automatic/semiautomatic segmentation methods developed for meningiomas.

Automatic grading of meningiomas using deep-learning technology has been reported in previous research [22,23,24,25]. We introduced the second deep-learning model with the classic DenseNet architecture in the third module, which was proposed as an excellent network for classification tasks in medical image datasets. DenseNet architecture was highlighted for the design of a dense block, in which each layer collected all feature maps from all preceding layers and passed on its own outputs as “collective knowledge” to all subsequent layers to preserve the feed-forward nature. It strengthens feature propagation, encourages feature reuse, and alleviates the vanishing-gradient problem [15]. Our research suggested that the DenseNet architecture network exhibited excellent performance with an AUC of 0.996 and accuracy of 0.969 in the validation group when classifying the tumors into low-grade and high-grade meningiomas. It should be noted that the improvements in classification accuracy were significant when the inputs were segmented images rather than completed images. The segmentation eliminated redundant information unrelated to the tumor image and encouraged feature extraction to be conducive to classification. When this module was integrated with the previous two modules, an intelligent system with a cascade structure was established for meningioma assessment.

The current study had several limitations. First, the system was built using deep-learning technology, but all patients were involved in the training set from a single institution. A large-scale multicenter study is required in the future. Second, the CNN architectures used in the research were all classic without too much modification, and we believe more novel network architectures should be explored in the future. Third, other intellectual tasks, such as brain cancer classification, should be integrated into the system in future research.

## Figures and Tables

**Table 1:** Characteristics of patients involved in the experimental dataset.

	Low-Grade Meningioma	High-Grade Meningioma
Number	500	125
Mean Age	49.38	53.24
Gender	Male: 131/500	67/125
	Female: 369/500	58/125
Days between MR scan and surgery	8.4 Days	7.6 Days

## Data Availability

Data are not available on request due to privacy and ethical restrictions.

## Supplementary Materials

The following are available online at https://www.mdpi.com/article/10.3390/jpm11080786/s1, Supplementary Figure S1: Examples in which the U-Net models did not perform well. A. tumors adjacent to thickening dura mater; B. tumors located in the anterior skull base; C. high-grade tumors with apparent necrosis. Supplement Figure S2: Bland–Altman plots for differences in tumor area.
