# Supplementary figures and images for: Automatic Meningioma Segmentation and Grading Prediction: A Hybrid Deep-Learning Method
Source: J Pers Med. 2021 Aug 12;11(8):786. doi: 10.3390/jpm11080786 (PMC8401675; doi:10.3390/jpm11080786)

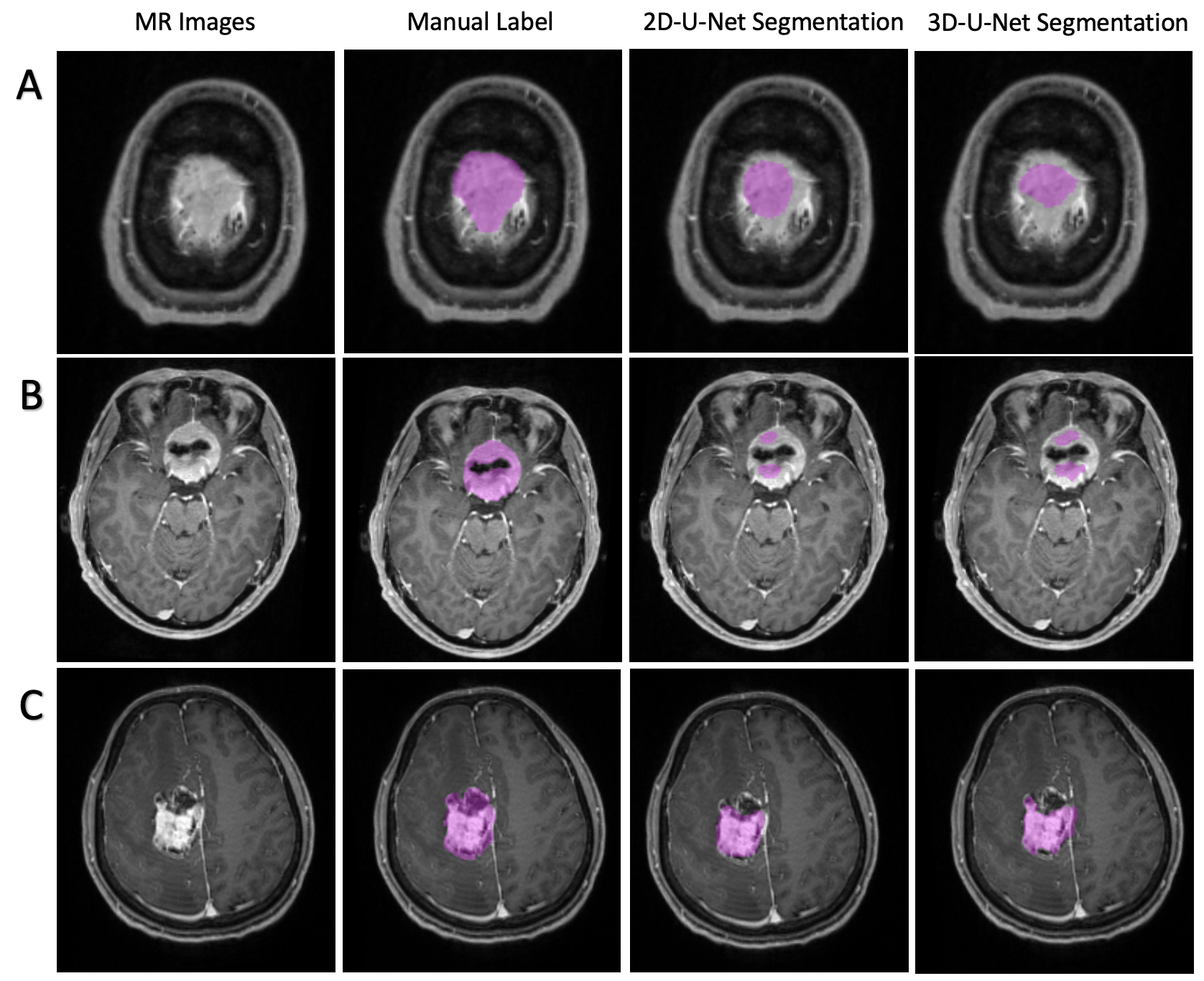

Supplement: Supplementary file 1 [file jpm-11-00786-s001.zip › SF.tif]

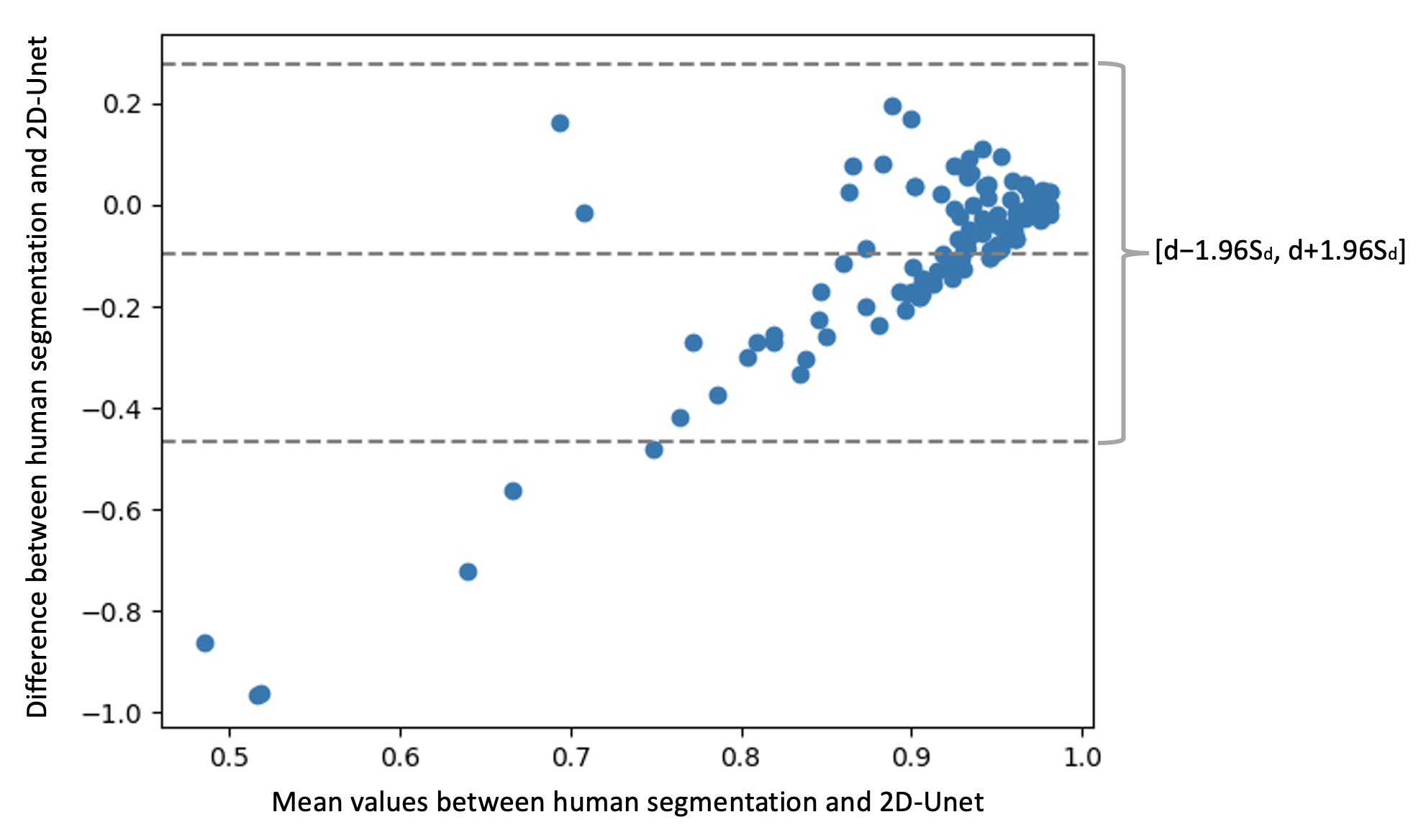

Supplement: Supplementary file 1 [file jpm-11-00786-s001.zip › SF2.tif]
